# Childhood adversity predicts worse verbal and visual memory in cognitively intact older White and Latino adults in California

**DOI:** 10.1002/alz.086262

**Published:** 2025-01-03

**Authors:** Miwa Tucker, Caitlin Wei‐Ming Watson, Valentina E. Diaz, Gloria A Aguirre, Jorge Achila Puac, Luis E Martinez, Anne‐Marie Rodriguez, Oscar Robles Archila, Charles Windon, Ashley J. Jackson, Stefanie D Pina‐Escudero, Joel H. Kramer, Serggio Lanata

**Affiliations:** ^1^ Memory and Aging Center, UCSF Weill Institute for Neurosciences, University of California, San Francisco, San Francisco, CA USA; ^2^ Memory and Aging Center, Weill Institute for Neurosciences, University of California, San Francisco, San Francisco, CA USA

## Abstract

**Background:**

Emerging research suggests adverse childhood experiences (ACEs) have long‐lasting impacts on adult brain health, but few studies investigate these effects in older adults. The present study examined ACEs and their relationships to late‐life cognitive and mental health among older adults living in the San Francisco Bay Area.

**Method:**

102 cognitively unimpaired older adults [*mean* age = 75, 58% female, 75% White, 25% Latino, *mean* education = 17 years] were enrolled in UC San Francisco’s Alzheimer’s Disease Research Center. Participants completed cognitive measures, the geriatric depression scale, and an ACEs survey (*threat ACEs*: e.g., abuse, discrimination; *deprivation ACEs*: e.g., unmet food or healthcare needs, neglect). Kruskal‐Wallis tests examined ACEs by sex and ethnicity. Linear regression models examined the effects of threat and deprivation ACEs on cognitive function and depression symptoms controlling for age, sex, and ethnicity. Sex and ethnicity were examined as potential moderators of these relationships.

**Result:**

On average, older adults reported 2.6 threat ACEs (*range* = 0‐12) and 0.8 deprivation ACEs (*range* = 0‐6). Females and males reported similar levels of ACEs. Latinos endorsed significantly more threat ACEs (^2^(1) = 6.0, *p* = 0.01) and deprivation ACEs (^2^(1) = 6.6, *p* = 0.01) compared to Whites. Older adults who reported more deprivation ACEs performed significantly worse on verbal memory (β = ‐0.30; *p* = 0.006) and visual memory tasks (β = ‐0.26, *p* = 0.04) while controlling for age, sex, and ethnicity. No significant relationships were observed between deprivation and executive functioning, object naming, visual‐spatial construction, or depression symptoms (*ps*>0.50), nor between threat ACEs and any outcome (*ps*>0.10). Sex and ethnicity did not moderate relationships between ACEs and cognitive and mental health outcomes.

**Conclusion:**

Our results suggest that deprivation ACEs may negatively and selectively impact the domain of memory among cognitively unimpaired older adults. Given episodic memory deficits are an early symptom of Alzheimer’s disease, this lowered memory function may increase dementia risk. Together, these findings suggest that early life interventions to support the basic needs of children could potentially reduce memory problems in late life. In future studies, cultural differences in the presentation and impacts of ACEs on late‐life cognitive and mental health should be examined to inform culturally adaptive interventions to support brain health.

